# Neurofibromatosis Type 1: Genetic Mechanisms and Advances in Therapeutic Innovation

**DOI:** 10.3390/cancers17233788

**Published:** 2025-11-26

**Authors:** Yuqing Lu, Manzhu Xu, Xiaojun Chen, Huazhen Xu, Nihao Sun, Karis E. Weisgerber, Ren-Yuan Bai

**Affiliations:** 1 Kennedy Krieger Institute, 707 N Broadway, Baltimore, MD 21205, USA; ylu146@jh.edu (Y.L.); mxu62@jh.edu (M.X.); xchen277@jh.edu (X.C.); hxu99@jh.edu (H.X.); kweisge1@jhu.edu (K.E.W.); 2 Department of Neurosurgery, Johns Hopkins University School of Medicine, 707 N Broadway, Baltimore, MD 21205, USA; 3 Department of Biomedical Engineering, Johns Hopkins University School of Medicine, 733 N Broadway, Baltimore, MD 21205, USA; nsun5@jh.edu

**Keywords:** neurofibromatosis type 1 (NF1), neurofibromin, *NF1* gene, plexiform neurofibromas (pNFs), malignant peripheral nerve sheath tumors (MPNSTs), MEK inhibitor, AAV-based gene therapy, GAP-related domain (GRD), oncolytic herpes simplex virus (oHSV) therapy, chimeric antigen receptor T cell (CAR-T) therapy

## Abstract

Mutations in the *NF1* gene cause Neurofibromatosis Type 1 (NF1), one of the most common genetic disorders. This gene encodes neurofibromin, a member of the GTPase-activating protein (GAP) family that functions as a negative regulator of RAS signaling. Loss of *NF1* function leads to persistent RAS activation and promotes tumor growth. The clinical manifestations of NF1 mainly include pigmentary changes, benign and malignant peripheral nerve sheath tumors, as well as gliomas affecting the central nervous system. Currently, MEK inhibition is the only approved therapy and is primarily effective in controlling plexiform neurofibromas (pNFs). However, more comprehensive treatments are needed to address the full spectrum of NF1 manifestations and malignant transformation. Novel therapeutic strategies, including AAV-based gene therapy aimed at restoring *NF1* function, oncolytic herpes simplex virus (oHSV) therapy targeting RAS-dysregulated tumor cells, and chimeric antigen receptor T cell (CAR-T) therapy targeting NF1-associated tumors, are under active investigation. In this review, we explore the genetic mechanisms underlying NF1 and highlight recent advances in therapeutic development with a special focus on AAV-based gene therapies alongside other approaches with recent clinical and translational advancements.

## Simple Summary

Neurofibromatosis type 1 is a common genetic disorder caused by loss-of-function mutations in the *NF1* gene. *NF1* deficiency drives constitutive RAS signaling and manifests a broad spectrum, from pigmentary changes to malignant peripheral nerve sheath tumors. Currently, the only approved therapy is MEK inhibition, which targets a limited subset of phenotypes and is not curative. In this review, we summarize NF1-driven signaling and emerging therapies, including AAV-based gene therapy, oHSV therapy, CAR-T cell therapy, and other molecular-targeted approaches. We emphasize recent progress in AAV-mediated delivery of the GAP-related domain using engineered capsids with Schwann-cell tropism. We discuss the advantages, the problems these strategies address, and their limitations. Understanding these evolving therapies could guide the development of improved therapeutic methods, enable rational combinations, and ultimately advance toward more comprehensive disease control.

## 1. Introduction

Neurofibromatosis type 1 (NF1) is an inherited disorder transmitted in an autosomal dominant manner characterized by a broad spectrum of manifestations. NF1 has an estimated global prevalence of approximately 1 in 3000 individuals, with around 50% of cases arising from de novo mutations, making it one of the most prevalent monogenic diseases [1]. Individuals with NF1 have significantly reduced life expectancy, typically dying on average 20 years earlier than the general population, with a notably shorter lifespan observed in women compared to men [2,3].

The *NF1* gene is located on chromosome 17q11.2. This gene encodes neurofibromin, a tumor suppressor protein that negatively modulates RAS signaling, thereby regulating cell growth and differentiation. Loss of neurofibromin function leads to persistent RAS activation, disrupting multiple signaling pathways and driving uncontrolled cellular proliferation, tumorigenesis, and disease progression [4].

Clinically, NF1 presents with a broad spectrum of manifestations, including pigmentary lesions, benign tumors such as cutaneous neurofibromas (cNFs) and plexiform neurofibromas (pNFs), and malignant tumors, particularly malignant peripheral nerve sheath tumors (MPNSTs) and gliomas affecting the central nervous system. These malignancies significantly contribute to morbidity and mortality in NF1 patients. Additionally, NF1 often causes cognitive impairment and impacts multiple organ systems, including cardiovascular, gastrointestinal, and musculoskeletal systems, and predisposes patients to other malignancies [5,6].

Despite recent advancements in understanding NF1 pathogenesis, therapeutic options remain limited, primarily relying on surgical resection and MEK inhibitors such as selumetinib and mirdametinib [7,8]. However, these treatments do not adequately address the broad therapeutic needs of all NF1 patients, particularly in preventing malignant transformation and achieving disease cure, and may lead to treatment resistance [9]. Therefore, there is an urgent need to investigate novel therapeutic strategies for effectively managing and treating the diverse clinical manifestations associated with NF1. This review aims to comprehensively discuss the molecular mechanisms underlying NF1 pathogenesis, evaluate current therapeutic options, and explore promising emerging therapies.

## 2. Molecular Mechanisms of NF1 Pathologies: Genetics and Pathways

### 2.1. The NF1 Gene

The *NF1* gene, located on chromosome 17q11.2, is one of the largest human genes, spanning approximately 350 kilobases and comprising approximately 61 exons [10]. Of these, 57 are constitutively expressed along with four exons (9a, 10a-2, 23a, and 48a) that are alternatively spliced to generate multiple transcript isoforms [11].

The entire *NF1* gene is located within a region of high linkage disequilibrium and low recombination, resulting in a relatively simple and conserved haplotype structure [12]. Despite this stable germline inheritance, *NF1* is among the most mutation-prone genes in the human genome [13], primarily due to its extensive size and possibly the presence of AT-rich sequences that facilitate retrotransposon insertions [14,15]. *NF1* also exhibits marked mutational heterogeneity with no well-defined mutation hotspots [16]. From the Human Gene Mutation Database (HGMD^®^), over 3000 *NF1* mutations have been reported, including missense and nonsense mutations, splicing defects, small insertions and deletions, as well as gross deletions. Notably, over 80% of *NF1* mutations identified in patients result in truncated neurofibromin proteins, indicating that loss-of-function is the leading cause of NF1 [12]. Similar to other tumor suppressor genes, *NF1* inactivation typically follows a biallelic “two-hit” model, where individuals inherit a germline mutation in one allele and acquire a somatic mutation in the second allele during early development or later in life, leading to localized tumor formation [17,18].

### 2.2. Neurofibromin and Signaling Pathway

Neurofibromin is a member of the GTPase-activating protein (GAP) family, with multiple isoforms generated by alternative splicing [19]. Skipping of exon 23a generates *NF1* isoform 1, predominantly expressed in neurons and characterized by higher RAS-GAP activity, whereas inclusion produces isoform 2 with lower RAS-GAP activity [20]. Insertion of exon 48a generates *NF1* isoform 3, while inclusion of both exons 23a and 48a produces isoform 4; both are expressed in heart muscle tissue [21]. Other isoforms include variants containing exon 9a, which are involved in neuronal maturation [22], and isoforms containing exon 10a-2, associated with localization to intracellular membranes [23]. Additionally, the *NF1* delta E43 isoform lacks exon 43, which encodes a nuclear localization signal (NLS), and is expressed at lower levels in neurons, indicating the importance of neurofibromin’s nuclear function in neurons [24]. In addition, studies have shown that full-length neurofibromin forms a high-affinity homodimer [25], and cryo-EM analyses have revealed two major dimeric assemblies, an autoinhibited closed state and an asymmetric open state, in which conformational changes regulate GRD access to RAS [26,27]. These findings indicate that proper dimerization is critical for neurofibromin function.

Neurofibromin comprises multiple functional domains, including the cysteine/serine-rich domain (CSRD), a tubulin-binding region (TBD), the GAP-related domain (GRD), a leucine-rich domain (LRD), the Sec14-like lipid-binding module, a pleckstrin homology (PH) domain, a C-terminal domain (CTD), a nuclear localization signal (NLS), and a syndecan-binding region (SBR) [19,28].

Starting from the N-terminal end, the CSRD can be phosphorylated by PKC upon RTK stimulation, facilitating interaction with actin and allosteric regulation of Ras-GAP activity [29]. Adjacent to the CSRD, the TBD mediates interactions with tubulin and promotes neurofibromin dimerization [25,30]. The GRD, comprising approximately 350 amino acids, is the most extensively studied domain, critically involved in negatively regulating RAS signaling by catalyzing the conversion of active Ras-GTP into inactive Ras-GDP [31]. This domain is highly conserved among RasGAP proteins such as p120-RasGAP and synaptic RasGAP (SynGAP) [32]. Following the GRD, the LRD contributes specifically to cell invasion independently of RAS signaling, as it lacks Ras-GAP activity and does not significantly affect proliferation [33]. Positioned downstream of the GRD’s C-terminal end, the Sec and PH domains are connected via a helical linker. The Sec domain serves as a lipid-binding module, with its activity regulated by conformational shifts within the PH domain [34]. Lastly, the CTD contains an NLS and an SBR. The NLS mediates nuclear localization and spindle association [35], whereas the SBR binds to syndecan proteins, influencing cell adhesion and migration [36].

**Figure 1:**
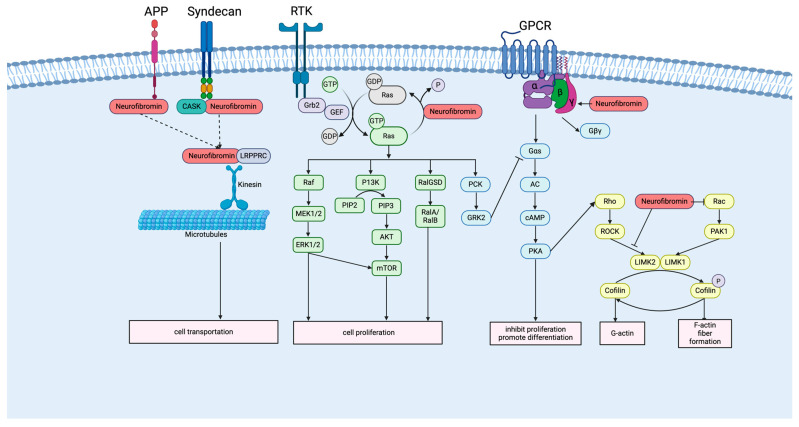
Neurofibromin-related signaling pathways. Loss of neurofibromin function drives persistent Ras activation (PI3K/AKT/mTOR, Ral, Raf/MEK/ERK), reduces cAMP from GPCR- and Ras/PKC-regulated pathways, and disrupts actin (Rho/ROCK/LIMK2, Rac1/PAK1/LIMK1) and microtubule transport (LRPPRC/kinesin), collectively promoting abnormal cell proliferation and differentiation, and cellular dysfunction. (Created in BioRender. Lu, Y., (19 November 2025) https://BioRender.com/5324mp8).

The *NF1*-deficient condition results in persistent RAS activation, causing hyperactivation of several oncogenic pathways, including the PI3K/AKT/mTOR (PAM) signaling pathway [37], the Ral signaling pathway [38], and the Raf/MEK/ERK signaling pathway (Figure 1) [39]. These dysregulated pathways promote abnormal cell proliferation, enhanced survival, and metabolic reprogramming, contributing to NF1-associated tumorigenesis [40]. Neurofibromin also positively regulates adenylyl cyclase (AC) activity (Figure 1) [41] through two distinct mechanisms: a RAS-independent pathway involving the activation of G protein-coupled receptors (GPCR), such as the 5-hydroxytryptamine receptor 6 (5-HT6r), via the PH domain, leading to increased Gαs/AC/cAMP signaling [42]; and a RAS-dependent pathway, where RAS activates PKC, resulting in the inhibition of downstream Gαs and cAMP production [43]. Both pathways, when disrupted by *NF1* deficiency, ultimately lead to reduced cAMP levels, resulting in abnormal cell proliferation and impaired neuronal functions, such as memory deficits [44].

Aside from neurofibromin’s function in regulating the RAS signaling pathway and cAMP activity, it also plays a critical role in regulating actin cytoskeleton organization and microtubule transport (Figure 1) [45]. It regulates actin cytoskeleton organization through two pathways: one involving Rho/ROCK/LIMK2/cofilin and another involving Rac1/Pak1/LIMK1/cofilin. The Sec-PH domain in neurofibromin can inhibit the LIMK2 phosphorylation through upstream ROCK [46], and the N-terminal extremity of neurofibromin can inhibit the Rac1/Pak1/LIMK1/cofilin pathway [47]. Both pathways, when disrupted by *NF1* deficiency, ultimately lead to the inactivation of cofilin and the formation of stress fibers. Neurofibromin also regulates microtubule-dependent transport by interacting with motor proteins, such as kinesin, and cargo-associated proteins, including leucine-rich pentatricopeptide-repeat-containing protein (LRPPRC), thereby modulating transport efficiency [30]. Additionally, studies have demonstrated that neurofibromin binds syndecans together with CASK [48] and can form complexes with amyloid precursor protein (APP) [49]. These interactions may function to localize neurofibromin to specialized domains within the plasma membrane and intracellular structures, influence protein trafficking, and affect synaptic plasticity and neuronal learning.

## 3. Clinical Manifestations of NF1

NF1 is characterized by diverse clinical features affecting multiple organ systems [50]. Cutaneous features typically emerge early, including pigmentary lesions such as café-au-lait macules (CALMs), axillary or inguinal freckling, and ocular findings such as Lisch nodules. CALMs and Lisch nodules often present in infancy or early childhood and progressively develop in nearly all patients with NF1. cNFs arise predominantly due to loss of heterozygosity (LOH) of the *NF1* gene within Schwann cells, affecting superficial nerves and generally remaining benign [51]. In the peripheral nervous system, pNFs originate from embryonic Schwann cell precursors and typically grow within deep nerve structures, extensively infiltrating surrounding tissues. This infiltration frequently results in significant functional impairment, pain, and severe disfigurement. Although cNFs and pNFs both arise from biallelic *NF1* loss and have near-identical histology, they differ markedly in NF1-related signaling pathways, tumor behavior, and malignant potential [52]. Recent epigenetic studies have shown that cNFs and pNFs harbor distinct DNA methylation patterns and higher-order chromatin organization, indicating that epigenetic mechanisms contribute to NF1 manifestations in addition to classical germline and somatic mutations [53]. Importantly, approximately 8–13% of pNFs undergo malignant transformation to MPNSTs, an aggressive sarcoma associated with poor prognosis [54]. Malignant transformation to MPNST is typically accompanied by additional somatic mutations beyond biallelic *NF1* inactivation. Homozygous loss of the *CDKN2A* locus occurs frequently, leading to inactivation of the tumor suppressors p14ARF and p16INK4A [55]. In parallel, inactivation of the polycomb repressive complex 2 (PRC2) through recurrent *SUZ12* and *EED* mutations is also common and results in loss of the repressive histone mark H3K27me3 [56]. Central nervous system manifestations commonly include optic pathway gliomas, typically arising in early childhood and potentially causing visual impairment or loss, along with low-grade pilocytic astrocytomas and other asymptomatic low-grade brain tumors [57]. Additionally, cognitive and behavioral difficulties, including learning disabilities, attention deficits, and challenges in social adaptation, occur with notable frequency in NF1 patients, significantly impacting quality of life [58]. Moreover, individuals with NF1 exhibit an increased incidence of various other malignancies, including breast cancer [59], rhabdomyosarcoma [60], and hematologic malignancies such as juvenile myelomonocytic leukemia [61]. Patients with NF1 also exhibit various skeletal abnormalities, such as nondystrophic scoliosis, bone dysplasia, and osteoporosis [62], as well as cardiovascular manifestations, including hypertension and increased risk of stroke [63]. These diverse features underscore the systemic involvement and clinical complexity of NF1.

## 4. Current Therapy for NF1

### 4.1. Surgical Management

**Figure 2:**
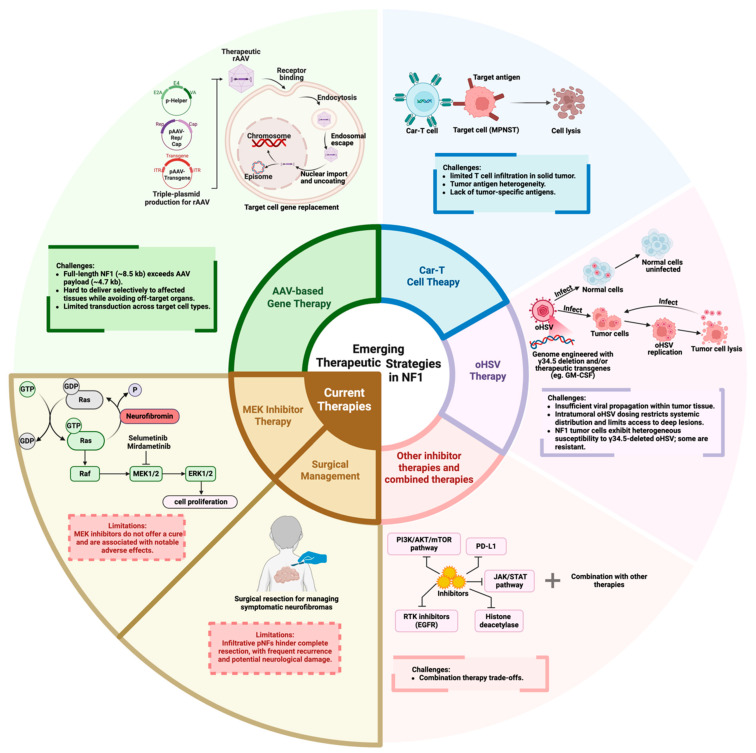
Therapeutic landscape for NF1-associated tumors. This figure summarizes current therapies, including surgical management and MEK inhibitor therapy, and their limitations. The figure also presents novel therapeutic strategies, including AAV-based gene therapy aimed at restoring NF1 function, oHSV therapy targeting RAS-dysregulated tumor cells, CAR-T cell therapy targeting NF1-associated tumors, and other inhibitor therapies, and outlines the challenges associated with each. (Created in BioRender. Lu, Y., (19 November 2025) https://BioRender.com/2tmit80).

Despite recent advances in understanding NF1-associated tumors, treatment options remain limited. Surgical resection remains the primary approach for managing symptomatic neurofibromas. Although surgery can provide symptom relief, complete removal is often challenging due to the infiltrative nature of tumors such as pNFs, and comes with a high risk of recurrence and potential damage to the nervous system (Figure 2) [64].

### 4.2. MEK Inhibitor Therapy

As neurofibromin functions as a negative regulator of RAS, pharmacological therapies for NF1 primarily target dysregulated RAS signaling, particularly the RAF/MEK/ERK pathway [54]. Selumetinib and mirdametinib are the only two MEK inhibitors approved for treating NF1. Selumetinib is FDA-approved for symptomatic, inoperable pNF in pediatric patients and, based on the phase 3 KOMET trial in adults, has also been approved for adult NF1 patients with symptomatic, inoperable pNF in Europe and Japan [7,65]. Mirdametinib is FDA-approved for both adult and pediatric patients with symptomatic pNF not amenable to complete resection [8]. Clinical trials have shown that both selumetinib and mirdametinib significantly reduce pNF volume, relieve pain, and stabilize tumor progression [8,66]. However, while effective in controlling tumor growth with continuous dosing in the majority of patients, MEK inhibitors do not offer a cure and are associated with notable adverse effects, including papulopustular rash, xerosis, and pruritus (Figure 2) [67].

## 5. Emerging Therapeutic Strategies

Given the limitations of current treatments, extensive research efforts have been directed toward developing novel therapeutic strategies for NF1. Among gene therapies, approaches include gene editing, such as CRISPR-based genome editing; gene replacement via vector-mediated delivery of the functional *NF1* gene [68], and splice-modulating approaches such as exon skipping [69]. Because the high mutational diversity across the *NF1* gene poses challenges for base-to-base gene-editing approaches, CRISPR-Cas9 is mainly used as a tool for gene-function studies and disease modeling in the NF1 field [70], and with only early therapeutic programs under development [71,72]. Exon-skipping strategies, most notably exon 17 or exon 52 skipping, aim to remove a mutation-containing exon while preserving the reading frame and can enable expression of a partially functional neurofibromin protein. It is a promising strategy that applies to selected patient subsets with pathogenic variants located in skippable exons [69,72]. Recently, the F-box protein FBXW11 has been identified as a regulator of neurofibromin degradation and can be potentially targeted to elevate neurofibromin levels in *NF1*^+/−^ individuals [73]. Overall, the delivery of functional *NF1* gene segments provides the opportunity to cover most forms of *NF1* loss. Common gene delivery vectors include viral systems such as adeno-associated virus (AAV), retrovirus, herpes simplex virus (HSV), and lentivirus, as well as non-viral platforms like plasmid DNA, lipid nanoparticles, and other nanoparticles [74,75]. Retroviruses and lentiviruses, as well as AAV, have been widely studied for NF1 gene delivery. However, in vivo use of retroviruses and lentiviruses is severely limited by the potential risk of insertional mutagenesis. AAVs offer a favorable safety profile and also the ability to transduce non-dividing cells [76]. Therefore, this review places particular emphasis on AAV-based gene therapy (Figure 2). In addition to gene therapy, other innovative approaches such as chimeric antigen receptor T cell (CAR-T) therapy, oncolytic herpes simplex virus (oHSV) therapy, and other molecular targets and pathway inhibitors are also being explored for their potential in treating NF1-related tumors (Figure 2). We also summarize the major preclinical and clinical advances in NF1-associated tumor therapy, including AAV-based gene delivery, oHSV approaches, CAR T-cell strategies, and small-molecule and pathway-targeted inhibitors (Table 1).

### 5.1. AAV-Based Gene Therapy

AAVs are widely used gene delivery vectors due to their low immunogenicity and inherent lack of pathogenicity. Additionally, AAVs can transduce non-dividing cells and persist as circular double-stranded episomal DNA within the nucleus, enabling long-term gene expression with minimal risk of insertional mutagenesis [86]. For NF1 gene therapy, research primarily focuses on AAV-mediated gene replacement therapy using recombinant adeno-associated viruses (rAAVs), which aims to restore functional neurofibromin expression by directly delivering a functional *NF1* gene into affected tissues, potentially reversing pathological RAS signaling dysregulation. However, AAV-based gene therapy for NF1 faces several challenges, including the large size of the *NF1* gene (approximately 8.5 kb), which exceeds the ~4.7 kb packaging capacity of standard AAV vectors, limited transduction efficiency across multiple affected cell types, and difficulty achieving targeted delivery, collectively restricting its therapeutic effectiveness in NF1 applications (Figure 2) [87].

To address the loading capacity of AAV vectors, current therapeutic strategies primarily focus on delivering a critical segment of the *NF1* gene rather than the full-length sequence. Hiatt et al. first demonstrated that the GRD of neurofibromin is sufficient to restore normal cell growth and RAS signaling in *NF1*-deficient primary cells, including hematopoietic progenitors, fibroblasts, and mast cells. This finding supports the possibility of GRD-based gene therapy in NF1 [88]. Subsequent studies by Thomas et al. and Bodempudi et al. further confirmed that expression of NF1-GRD reduces RAS pathway activation and/or suppresses invasive behavior in *NF1*-deficient cells [89,90]. Later, Bai et al. provided critical translational evidence that AAV-mediated delivery of the NF1-GRD, particularly membrane-targeting variants fused with motifs such as the C-terminal 10 AA of HRAS domain (C10), the C-terminal 24 AA of KRAS4B (C24), and other similar RAS C-terminal domains, effectively suppressed aberrant RAS signaling and reduced tumor growth in NF1-associated MPNSTs [91]. While delivering only the GRD offers a practical solution to the packaging limitations of AAV vectors, concerns remain that *NF1* mutations are not limited to the GRD region but are distributed throughout the entire gene, which may affect the neurofibromin’s overall expression, structure, or regulatory functions [92]. An additional consideration for *NF1* gene replacement is how AAV-delivered neurofibromin will interact with endogenous mutant proteins that can form dominant-negative dimers [26]. In principle, GRD-based constructs are expected to be less sensitive to dominant-negative effects because they lack other dimerization components and have shown robust rescue of RAS signaling in preclinical NF1 models [91,93].

**Figure 3:**
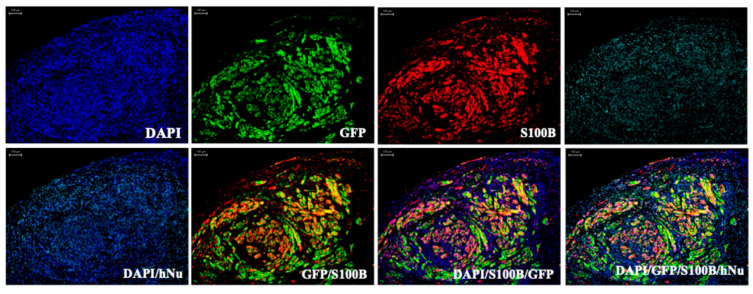
AAV-K55-GFP selectively transduces Schwann cell-like populations in an iPSC-derived neurofibroma xenograft. Immunofluorescence (IF) analysis of sections from a neurofibroma xenograft generated by mixing 3MM *NF1*^−/−^ Schwann cells with pNF-derived fibroblasts in 3D culture and engrafting onto the mouse sciatic nerve according to Mazuelas et al. [97]. After AAV-K55-GFP administration, tumor sections were triple-stained with antibodies against GFP (green), S100B (red), and human nuclear antigen (hNu) (cyan), with nuclei counterstained with DAPI (blue). The merged images show strong co-localization of GFP and S100B, indicating preferred transduction of Schwann cell-like populations by AAV-K55. The hNu staining confirms the human origin of tumor cells and indicates other cells of human origin, presumably pNF-derived fibroblasts. Scale bar = 100 µm.

Additionally, improving transduction efficiency and specificity has been a key focus of recent efforts in AAV capsid engineering for *NF1* gene therapy. Initial studies identified naturally occurring AAV serotypes with promising tropism, providing valuable foundations for subsequent engineering. Bai et al. demonstrated that AAV serotypes 1, 2, 3B, 6, and DJ efficiently transduced human Schwann cells (hSCs) and MPNST cell lines [91]. Additionally, Kagiava et al. and Tanguy et al. reported that AAV9 and AAVrh10 can effectively transduce Schwann cells throughout the peripheral nervous system [94,95]. Later, advances in rational design and directed evolution have enabled the generation of engineered AAV capsids with significantly enhanced and selective tropism [96]. For instance, Drouyer et al. developed two novel AAV capsids, Pep2hSC1 and Pep2hSC2, with enhanced and specific transduction efficiency in hSCs using a functional transduction-RNA selection method (Table 1) [77]. Similarly, Haidar et al. screened an AAV9-based peptide display library, resulting in AAV-SC3, which showed robust transduction efficiency in neurofibromas, and AAV-SC4, which targeted normal sciatic nerves via a laminin-binding motif and exhibited a 6-fold higher biodistribution compared to parental AAV9 (Table 1) [78]. In addition, our study engineered a novel AAV capsid, AAV-K55, through capsid DNA shuffling and in vivo peptide library screening in NF1 xenograft mice (Table 1). AAV-K55 showed significant therapeutic efficacy in human xenograft MPNST mouse models and exhibited strong Schwann cell tropism in human pNF-iPSC-derived neurofibroma (Figure 3). AAV-K55 markedly enhanced tumor selectivity, reduced liver uptake, and demonstrated significant therapeutic efficacy in delivering the GRD-C24 transgene across multiple NF1 tumor models, including pNFs, MPNSTs, and gliomas [79]. These customized AAV capsids offer improved targeting capabilities, minimizing off-target effects and immunogenic responses, thereby providing safer and more effective platforms for future systemic AAV delivery strategies in NF1 therapy.

To further overcome the packaging limitation of AAV vectors, novel techniques such as split AAV vector systems and oversized AAV vectors are being explored. Split AAV systems enable delivery of large genes by dividing the transgene across multiple vectors, which reconstitute the full-length protein through DNA recombination, RNA trans-splicing, or intein-mediated protein ligation [98]. While this approach has not yet been applied directly to *NF1* gene delivery, recent work performed by Maddalena et al. demonstrated the feasibility of using a triple AAV system to express the full-length ALMS1 gene (12.5 kb) successfully in the retina, which is significantly larger than the *NF1* coding sequence (~8.5 kb) [99]. Alternatively, oversized AAV vector strategies aim to enhance packaging capacity by optimizing vector assembly, stability, and intracellular processing. Recent research showed that proteasome inhibitors can partially mitigate proteasomal degradation, enabling encapsulation of genetic payloads up to approximately 6.0 kb [100]. However, current oversized vector approaches remain limited in capacity and still pose challenges for delivering large genes such as *NF1*.

Overall, while AAV-mediated gene therapy for NF1 is still in the developmental stages, significant advances have been made in addressing the fundamental limitations, including loading capacity and targeted cell specificity. The feasibility of using GRD constructs combined with advanced engineered AAV vectors makes gene therapy a promising therapeutic approach. Future research focusing on the safety, specificity, and efficiency of these methods is called for further clinical translations.

### 5.2. CAR-T Cell Therapy

CAR-T cell therapy utilizes engineered T cells to target tumor-specific antigens and has been approved for years to treat myeloma and B-cell malignancies, demonstrating remarkable efficacy [101]. However, its application in solid tumors has been more challenging due to limited T cell infiltration, tumor antigen heterogeneity, and the lack of truly tumor-specific antigens. Despite these limitations, active research is addressing these barriers [102]. Recent CAR-T therapies for solid tumors have shown encouraging results. A phase 1 trial treating patients with advanced gastrointestinal cancers achieved a 38.8% overall response rate and a 91.8% disease control rate without DLT [103]. Another phase 1–2 clinical trial evaluated GD2-targeted CAR-T cells in children with relapsed or refractory high-risk neuroblastoma, demonstrating a 63% overall response rate, manageable toxicity, long-term CAR-T cell persistence, and a 60% three-year overall survival in patients receiving the recommended dose [104]. These trials support CAR-T as a durable immunotherapy strategy for solid tumors.

Although clinical application of CAR-T therapy for NF1 tumors is still at an early stage, multiple Phase I trials have recently begun evaluating its safety and feasibility in NF1-associated malignancies, particularly MPNST. A Phase I study (NCT03618381) is testing EGFR806-directed CAR-T cells in pediatric and young adult patients with relapsed or refractory EGFR-expressing solid tumors, including NF1-associated MPNST. Two additional Phase I trials (NCT04483778 and NCT04897321) are investigating B7-H3-targeted CAR-T cell therapies in patients with B7-H3-positive solid tumors, also enrolling individuals with NF1-associated MPNST, including bispecific B7-H3xCD19 CAR-T constructs and combination therapy with pembrolizumab to enhance antitumor activity and persistence. Moreover, a Phase I/II trial (NCT04085159) is evaluating personalized antigen-specific CART/CTL therapy combined with dendritic-cell vaccination in patients with neurofibromatosis (NF1, NF2) or schwannomatosis and progressive NF-related tumors. Collectively, these early-phase trials represent pioneering efforts to translate CAR-T approaches into NF1-associated disease and provide an emerging clinical foundation supporting future development (Table 1).

In addition to these clinical developments, the feasibility of CAR-T approaches for NF1 tumors has been comprehensively evaluated by Tang et al., who identified HER1 as a promising CAR-T target in NF1-associated nerve sheath tumors. They demonstrated that anti-HER1 CAR-T cells, particularly those using the 806 scFv, effectively eliminated tumor cells in vitro. The efficacy was further enhanced by TGFBR2 and PDCD1 knockout, which helped overcome the immunosuppressive tumor microenvironment [105]. Together, these early clinical and experimental studies highlight CAR-T therapy as a promising strategy for NF1-related tumors.

### 5.3. oHSV Therapy

oHSVs are derived from HSV-1 and genetically modified to replicate selectively in tumor cells while sparing normal tissue. These vectors can also be armed with therapeutic transgenes to enhance tumor-specific cytotoxicity or stimulate antitumor immunity [106]. Several oHSV variants are currently under investigation in clinical and preclinical settings, including T-VEC [107], HSV1716 [108], and G47Δ [109]. Among them, T-VEC is the only FDA-approved oHSV for the treatment of unresectable melanoma [110].

A common feature of many oHSV variants is the deletion of the γ34.5 gene, which encodes ICP34.5, a protein that helps the virus counteract host antiviral defenses by recruiting protein phosphatase 1α (PP1α) to dephosphorylate eIF2α, thereby restoring protein synthesis in infected cells [111]. In normal cells, HSV-1 infection activates the PKR pathway, leading to eIF2α phosphorylation and shutdown of protein synthesis as an antiviral response. Deletion of γ34.5 renders the virus replication-deficient in normal cells but allows selective replication in tumor cells where the PKR activation is suppressed [112]. Notably, several human tumor cell lines, such as HT1080 and PANC-1, are permissive to γ34.5-deleted oHSV due to constitutive activation of the MEK, which suppresses PKR autophosphorylation and allows continued protein synthesis [113].

Since *NF1*-deficient tumors exhibit hyperactive RAS/RAF/MEK signaling, they also represent ideal candidates for oHSV therapy. Early preclinical studies using oHSV G47Δ in NF1-associated MPNST models have demonstrated significantly inhibited tumor growth and prolonged survival [114]. Further strategies to improve efficacy include arming oHSVs with immunomodulatory genes or antiangiogenic factors. For instance, Liu et al. engineered G47Δ to express a dominant-negative FGFR (bG47Δ-dnFGFR), which significantly enhanced its antitumor and antiangiogenic efficacy in NF1-associated neural tumors by simultaneously targeting tumor cells and tumor vasculature, without impairing viral replication [115]. These encouraging findings, together with the growing availability of clinically relevant NF1 and MPNST models, suggest that oHSV-based therapies hold strong potential for future MPNST treatment and clinical translation [116].

Building on these preclinical advances, multiple early-phase clinical trials have now begun evaluating oHSV platforms in patients with NF1-associated tumors. A Phase I study (NCT07102394) is assessing intralesional IMLYGIC (T-VEC) monotherapy for cNFs in adults with NF1, with feasibility defined by completion of four 28-day treatment cycles and safety evaluated through DLT and lesion-response assessments. Another Phase I trial (NCT00931931) is evaluating HSV1716 delivered intratumorally or intravenously in adolescents and young adults with refractory non-CNS solid tumors, including NF1-associated MPNST, with primary safety and DLT assessment and extended immune-monitoring planned for long-term follow-up. Furthermore, a Phase Ib/II study (NCT06660810) is examining neoadjuvant T-VEC combined with preoperative external-beam radiation for locally advanced unresectable soft tissue sarcoma, including NF1-associated MPNST, aiming to improve pathological response and survival outcomes. Together, these trials highlight increasing clinical commitment to oHSV platforms and support continued development of engineered oHSV approaches for NF1-related tumors (Table 1).

### 5.4. Other Molecular Targets and Pathway Inhibitors

Beyond the two FDA-approved MEK inhibitors, a range of additional targeted therapies are under active investigation for NF1-associated tumors, focusing on signaling pathways, epigenetic regulation, and immune modulation [117]. Downstream inhibition of MAPK signaling is also being explored through ERK blockade. Ulixertinib, a first-in-class ERK inhibitor, is currently being investigated in an early Phase I study (NCT05804227) enrolling adolescents and adults with MAPK-activated gliomas, including NF1-associated low-grade gliomas. This trial evaluates whether preoperative ulixertinib can penetrate the blood–brain barrier and modulate ERK pathway activity. In addition to monotherapy, ulixertinib is evaluated in combination with the CDK4/6 inhibitor palbociclib (NCT03454035), enrolling patients with advanced solid tumors, including an expansion cohort for metastatic RAS-mutant and NF1-mutant melanoma (Table 1). Within the RAS pathway, the pan-RAS inhibitor RMC-7977 demonstrated significant preclinical efficacy in NF1-related tumor models, including MPNST and glioma [118]. Another RAS-directed approach evaluated in patients is the farnesyltransferase inhibitor tipifarnib (R115777). In a randomized, double-blind, placebo-controlled Phase II trial (NCT00021541), tipifarnib was administered to children and young adults (3–25 years) with NF1-associated pNF. Although the regimen was well-tolerated, the study did not demonstrate a significant improvement in time to volumetric progression compared with placebo (Table 1). Direct RAS targeting may offer a more durable strategy than inhibiting downstream effectors, as resistance to MEK and ERK inhibitors commonly arises through activation of compensatory pathways [9]. In the PAM pathway, sirolimus and its derivative everolimus inhibit mTORC1 signaling and show significant preclinical efficacy in NF1-associated MPNST models [119]. However, a meta-analysis of four phase II trials found that everolimus did not significantly reduce NF1-associated lesion size and calls for further evaluation of its efficacy [81]. In the JAK/STAT pathway, the small-molecule inhibitor FLLL32 has shown the ability to suppress pNF growth by reducing inflammatory cytokine expression, macrophage proliferation, and Schwann cell survival [120]. cAMP modulators such as rolipram and forskolin have demonstrated neuroprotective effects by rescuing neurodevelopmental defects and reducing apoptosis in *NF1*^+/−^ CNS neurons [121]. Inhibitors targeting receptor tyrosine kinases (RTKs), including Platelet-Derived Growth Factor Receptor (PDGFR), VEGFR, MET, RET, and EGFR, are also under evaluation. Imatinib, a multitarget RTK inhibitor that blocks KIT and PDGFR signaling, is of particular interest in NF1 MPNSTs [122]. A Phase II clinical trial (NCT01673009) tested daily imatinib in children and adults with NF1-associated pNF, evaluating radiographic response and treatment tolerability, and supports the feasibility of imatinib mesylate as a targeted therapeutic approach (Table 1). Notably, MET amplification has been identified as a driver of MPNST progression in NF1, and tumors with this alteration exhibit strong sensitivity to the MET inhibitor capmatinib, especially when combined with MEK inhibition [123]. As an RTK inhibitor targeting MET and VEGFR2 [85], cabozantinib achieved partial responses in 42% of evaluable patients, producing a median tumor-volume reduction of 15.2% without any progression during treatment, while also demonstrating improvements in pain and quality-of-life measures alongside a manageable toxicity profile in a Phase II single-arm study of children with NF1-associated pNF (NCT02101736). Sorafenib, another multikinase inhibitor with activity against RAF, VEGFR, and PDGFR, is evaluated in a Phase I dose-escalation study (NCT00727233, Table 1) [84]. Beyond targeted inhibitors, mebendazole has shown chemopreventive effects against malignant transformation in NF1-associated tumor models, accompanied by reduced RAS activity [124]. In the immune checkpoint category, elevated PD-L1 expression has been observed in MPNST patients [125] and is reported to achieve a complete metabolic response to the PD-1 inhibitor pembrolizumab in a patient with PD-L1–positive metastatic MPNST [126]. A phase II trial of pembrolizumab in patients with unresectable or metastatic MPNST (NCT02691026) was initiated to evaluate objective response and safety; however, the study was terminated early, and results have not yet established a clear efficacy signal. Dual checkpoint blockade is also being explored: nivolumab, a PD-1 inhibitor, and ipilimumab, a CTLA-4 inhibitor, act synergistically by enhancing T-cell activation and reducing tumor-induced immunosuppression [127]. An early-phase neoadjuvant study (NCT04465643) is evaluating the safety and feasibility of administering nivolumab plus ipilimumab prior to surgical resection of NF1-associated atypical neurofibromas and MPNST (Table 1). In terms of epigenetic regulation, histone deacetylase inhibitors (HDACis) such as PCI-24781 have shown efficacy in inducing apoptosis and autophagy in *NF1*-associated MPNSTs, particularly in *NF1*-driven tumors [128]. Beyond HDACis, hypomethylating strategies are being explored for PRC2-deficient NF1-MPNST, where loss of epigenetic repression contributes to malignant progression [129]. ASTX727 (INQOVI) is a fixed-dose oral combination of cedazuridine, a cytidine deaminase (CDA) inhibitor, and decitabine, a DNA methyltransferase (DNMT) inhibitor. A Phase II open-label trial (NCT04872543) is evaluating the efficacy, safety, and tolerability of ASTX727 in adults and adolescents with PRC2-loss MPNST (Table 1). These agents represent promising candidates for future clinical use, either as monotherapies or in combination with other targeted therapies [130].

## 6. Conclusions and Perspectives

NF1 has long been and remains a challenging genetic disease because of the gene’s complexity, along with heterogeneous disease mechanisms, and variable clinical manifestations with a high rate of de novo cases. Recent progress in understanding the complex structure of the NF1 gene and the domain architecture and functions of neurofibromin has inspired the development of current targeted therapeutic strategies. Current therapeutic approaches mainly focus on the dysregulated RAS signaling pathway. The recently approved MEK-inhibition therapy targeting the RAS pathway, including selumetinib and mirdametinib, has revolutionized the traditional surgical management of NF1, although it is limited to pNF patients and is not curative, but it can stabilize tumor progression [7,124]. More therapeutic methods are called for other NF1 manifestations, especially for the prevention, control, and cure of malignant NF1 tumors.

More fundamental changes have been promised by gene therapy to restore functional neurofibromin. AAV, a widely used, safe gene delivery tool, has been widely explored in NF1 gene therapy recently. Because the full-length NF1 gene exceeds the single-AAV payload, current research prioritizes delivering the main functional domain of neurofibromin, especially the membrane-targeted GRD constructs [91]. And AAV capsid engineering is applied to improve delivery tropism towards Schwann cells, reduce off-target toxicity, and enhance therapeutic effect. AAV-mediated GRD gene delivery has demonstrated robust preclinical rescue of the RAS signaling pathway and therapeutic effects across various NF1 models, and it offers the potential to advance translational development [79]. As a promising field for future therapy, further preclinical validation and clinical trials are crucial and urgently required for broader applications.

Other emerging therapeutic methods, such as oncolytic virus strategies, also offer promising tools for targeted therapy. oHSV has its own advantages in various aspects; T-VEC has already been approved for melanoma as a safe and effective tool, and oHSV also shows strong preclinical effects in treating NF1 tumors [107,114]. Further clinical trials are urgently required for the expansion of oHSV usage in the NF1 field. In addition, oHSV can also be genetically modified to arm it with functional treatment genes, including immunomodulatory genes or antiangiogenic factors. With oHSV’s larger genome size, it enables a larger genetic capacity as a gene-delivery tool compared to AAV [131]. This makes oHSV promising as both an effective NF1 tumor oncolytic tool and a gene-delivery tool for effective treatment.

Other cellular and small-molecular strategies in NF1, including CAR-T cell therapy and molecular inhibition therapy, are also under investigation. Although CAR-T is challenged by its application barrier in solid tumors [132], especially in those intensive solid tumors like NF1, it holds its potential to gain favor in NF1 for systemic delivery and has no concern about antiviral antibodies compared to the use of AAV and oHSV, which is a common concern in viral-therapy treatment [133,134]. With future mechanistic studies on NF1 cells’ specific epitopes as targeting sites, CAR-T could be a powerful tool in NF1. In addition to CAR-T therapy, inhibitor therapy offers a more straightforward approach with the advantages of simplicity, safety, and established standard administration. Supported by extensive studies of complex NF1-related signaling pathways, beyond the approval of MEK inhibitors, small-molecule therapies targeting PAM, JAK/STAT, RTKs, epigenetic regulators, and other pathways related to NF1 are extensively studied [120,121]. For further translational applications, toxicity and resistance require further validation in preclinical and clinical settings.

Each emerging therapeutic strategy for NF1 has distinct strengths and limitations (Figure 1). Gene therapy approaches such as AAV-mediated *NF1* gene replacement aim to restore the genetic defect but face challenges related to limited AAV payload capacity, capsid tropism for achieving sufficient and selective transduction, pre-existing immunity, and long-term safety considerations. Oncolytic HSV relies on tumor-intrinsic permissiveness and often requires local injection, which restricts systemic distribution and limits access to deep lesions. CAR-T therapy faces challenges such as antigen heterogeneity, limited T-cell infiltration into solid tumors, and off-target effects. In addition, small-molecule inhibitors targeting RAS, PAM, RTKs, JAK/STAT, or epigenetic regulators frequently yield only partial responses as monotherapies and are limited by systemic toxicities. Collectively, we see the potential of rational combination therapies across the above-mentioned therapeutic methods, for example, combining AAV gene therapy with a MEK inhibitor or with oHSV therapy to improve therapeutic efficacy. However, these combination therapies also require further preclinical and clinical validation to enhance therapeutic effects and to carefully evaluate potential mechanistic trade-offs.

Moving forward, integrating these advanced therapies into clinical practice could treat more NF1 patients with diverse disease manifestations and cure or control the progression of malignant NF1 tumors. And this will finally enable more personalized, effective, and safe treatments that lead to prolonged patient survival and better quality of life for all NF1 patients.

## Figures and Tables

**Table 1:** Preclinical and Clinical Advances in Therapeutic Strategies for NF1-Associated Tumors.

Therapy	Specific Agent	Tumor Type	Status	Study Purpose	Treatment Plan	Reference
AAV Therapy	Pep2hSC1 and Pep2hSC2 capsids	pNF–derived Schwann cells (pNF01.3), C57BL/6J mice, hFRG mice (FRG mice engrafted with human hepatocytes)	Preclinical Study	This preclinical study investigates newly engineered AAV capsids, Pep2hSC1 and Pep2hSC2, to determine their capacity to efficiently and selectively transduce human Schwann cells, including those derived from NF1-associated pNF. Both vectors demonstrate substantially higher Schwann-cell tropism than existing AAV serotypes, with Pep2hSC2 showing exceptional specificity by avoiding fibroblast transduction.	16-week-old male C57BL/6J mice underwent sciatic nerve exposure, bilateral 10-s crush injury, and immediate microinjection of 3 µL AAV (AAV-DJ, Pep2hSC1, or Pep2hSC2) both proximal and distal to the crush site. 6 to 8-week-old hFRG mice were injected intravenously with 2 × 10^11^ vg of each AAV variant via the tail vein.	Drouyer et al. [77]
	AAV-SC3 and AAV-SC4 capsids	NF1 and Charcot-Marie Tooth disease involve SCs in C57BL/6 mice and *Nf1*^flox/flox^/*Fluc*^flox^ mice	Preclinical Study	This study is designed to develop and evaluate engineered AAV9 capsids—AAV-SC3 and AAV-SC—capable of efficiently targeting Schwann cells and NF1-associated neurofibromas after systemic delivery.	Different dosages of AAV were injected through the tail vein from 10^10^ vg to 10^12^ vg/mouse.	Haidar et al. [78]
	AAV-K55 capsid with GRDC24 as payload	NF1-related MPNST, pNF, neurofibromas, glioma xenografted in NSG mice	Preclinical Study	This study is designed to develop and evaluate the AAV-K55 capsid, capable of delivering a functional truncated neurofibromin construct, GRDC24, to NF1-associated tumors, with the goal of inhibiting RAS signaling and restoring Schwann-cell function.	Two separate 1 × 10^12^ vg doses or a single 2 × 10^12^ vg dose of AAV-GRDC24 were administered by tail-vein injection to xenografted NSG mice.	Bai et al. [79]
oHSV Therapy	IMLYGIC (Talimogene laherparepvec (T-VEC))	cNF in adults (≥18 yrs)	Phase I (single-arm, open-label, interventional)	This study is designed to evaluate the feasibility, tolerability, and efficacy of IMLYGIC for treating cNF in adults with NF1.	The trial administers IMLYGIC as an intralesional monotherapy delivered over four 28-day treatment cycles, with clinical evaluation throughout to monitor response.	NCT07102394
	HSV1716	Refractory non-CNS solid tumors, including NF1-associated MPNST in adolescents and young adults (7–30 yrs)	Phase I (single-arm, open-label, interventional, non-randomized)	This study is designed to assess the safety and dose-limiting toxicities (DLT) of HSV1716 administered either intratumorally or intravenously in patients with refractory solid tumors, including MPNST.	The trial delivers a single escalating dose with the option for additional dosing in a follow-up study phase, with toxicity assessments at 28 days and long-term immune monitoring for up to 15 years.	NCT00931931
	T-VEC	Locally advanced unresectable soft tissue sarcomas (STS), including NF1-associated MPNST in adults (≥18 yrs)	Phase Ib/II (single arm, open-label, interventional)	This study is designed to evaluate the efficacy and safety of combining neoadjuvant T-VEC with preoperative radiation to improve pathological tumor response in unresectable STS.	The trial administers weekly intratumoral T-VEC starting at week 1, concurrent daily external-beam radiation during weeks 4–8, and continued weekly T-VEC through weeks 9–12, followed by surgical resection 4–6 weeks post-radiation.	NCT06660810
CAR-T cell Therapy	Arm A: second-generation 4-1BBζ EGFR806-EGFRt CAR-T cells Arm B: second-generation 4-1BBζ EGFR806-EGFRt plus second-generation 4 1BBζ CD19-Her2tG CAR-T cells	Recurrent or refractory malignant non-CNS solid tumors expressing EGFR, including NF1-associated MPNST, in pediatric and young adult patients (1–30 yrs)	Phase I (two-arm, open-label, interventional, non-randomized, parallel-assignment)	This study is designed to evaluate the safety, manufacturability, DLT, and early antitumor activity of autologous EGFR806-based CAR-T products in pediatric and young adult patients with relapsed or refractory EGFR-expressing non-CNS solid tumors, including MPNST.	Patients undergo leukapheresis and receive a single infusion of CD4/CD8 EGFR806 CAR-T cells alone (Arm A) or dual EGFR806xCD19 CAR-T cells (Arm B), followed by intensive monitoring for toxicity, CAR-T cell persistence in blood and bone marrow, and changes in tumor burden.	NCT03618381
	Arm A: second-generation 4-1BBζ B7H3-EGFRt-DHFR CAR (B7H3-specific CAR-T cells). Arm B: second-generation 4-1BBζ B7H3-EGFRt-DHFR plus second-generation 4-1BBζ CD19-Her2tG (bispecific B7H3 × CD19 CAR-T cells). Arm C: same bispecific B7H3 × CD19 CAR-T cells as Arm B, given together with pembrolizumab.	Recurrent or refractory malignant non-primary CNS solid tumors expressing B7H3, including NF1-associated MPNST, in pediatric and young adult patients (0–26 yrs)	Phase I (three-arm, open-label, interventional, non-randomized, sequential-assignment)	This study is designed to evaluate the safety, feasibility, DLT, and maximum tolerated dose (MTD) of B7H3-directed CAR-T cell products, as well as their persistence, in children and young adults with relapsed or refractory non-CNS solid tumors, including MPNST.	The trial administers a single infusion of autologous B7H3 CAR-T cells alone (Arm A), bispecific B7H3 × CD19 CAR-T cells (Arm B), or bispecific B7H3 × CD19 CAR-T cells combined with pembrolizumab (Arm C), with serial assessments of toxicity, CAR-T persistence, and tumor response.	NCT04483778
	B7-H3-CAR-T cells post-lymphodepleting chemotherapy	Relapsed or refractory B7-H3-positive non-CNS solid tumors, including NF1-associated MPNST, in pediatric and young adult patients (≤21 yrs)	Phase I (single-arm, open-label, interventional)	This study is designed to evaluate the safety, DLT, and MTD of autologous B7-H3 CAR-T cells in children and young adults with relapsed or refractory B7-H3–expressing solid tumors, including MPNST.	The trial delivers lymphodepleting fludarabine/cyclophosphamide followed by a single weight-based infusion of B7-H3 CAR-T cells, with a 6-week DLT evaluation period and ongoing follow-up through one year before transition to institutional long-term monitoring.	NCT04897321
	Antigen-specific cytokine-activated T cells (CART)/cytotoxic T lymphocytes (CTLs) and dendritic cell vaccine (DCvac)	Neurofibromatosis (NF1, NF2) or schwannomatosis in patients (1–80 yrs)	Phase I/II (single-arm, open-label, interventional)	This study is designed to evaluate the safety and preliminary therapeutic activity of autologous CART/CTL plus DCvac immunotherapy in patients with neurofibromatosis or schwannomatosis who have progressing NF-related tumors.	The trial manufactures patient-specific antigen-reactive CART/CTL products and DCvac from autologous cells and delivers combined CART/CTL/DCvac infusions, with longitudinal monitoring of safety, tumor-associated markers, and radiographic response over 12–24 months.	NCT04085159
Small Molecule Inhibitors	Selumetinib (AZD6244 hyd sulfate) MEK Inhibitor	NF1-associated pNF patients aged 1 yr and older	FDA Approved	Selumetinib was first approved by the FDA on 10 April 2020, for pediatric NF1 PN patients aged 2 years and above based on the SPRINT Phase II Stratum I study (NCT01362803). On 10 September 2025, this approval was broadened to include patients as young as 1 year old, based on the SPRINKLE study (NCT05309668).	Selumetinib is recommended at 25 mg/m^2^ orally twice daily, given until progression or intolerable toxicity.	NCT01362803 NCT05309668
	Mirdametinib (PD-0325901) MEK Inhibitor	NF1-associated pNF patients aged 2 yrs and above	FDA Approved	Mirdametinib was approved by the FDA on 11 February 2025, for pediatric NF1 PN patients aged 2 years and above who are not amenable to complete surgical resection based on the ReNeu study (NCT03962543).	Mirdametinib is dosed at 2 mg/m^2^ orally twice daily for 21 days of each 28-day cycle, with or without food, and is continued until progression or intolerable toxicity.	NCT03962543
	Tipifarnib (R115777) RAS Inhibitor	NF1-associated pNF in children and young adults (3–25 yrs)	Phase II (Interventional, randomized, flexible crossover, double-blinded, placebo-controlled trial)	This study is designed to determine whether the farnesyltransferase inhibitor tipifarnib can delay volumetric progression of pNF and to characterize its safety profile in children and young adults with NF1. Tipifarnib was well-tolerated but did not significantly prolong time to progression versus placebo [80].	The patients receive oral tipifarnib or placebo at 200 mg/m^2^ twice daily on days 1–21 of repeated 28-day cycles, with clinical evaluation throughout to monitor response.	NCT00021541
	Ulixertinib ERK Inhibitor	NF1-associated low-grade glioma (LGG) (Surgical ≥ 18 yrs; Non-surgical ≥ 12 yrs)	Early Phase I (Interventional, parallel assignment, open-label)	This study is designed to determine whether ulixertinib can cross the blood–brain barrier and to assess the safety and biological effects of preoperative ulixertinib in MAPK-driven gliomas, including NF1-associated LGG.	Participants will receive ulixertinib at the recommended phase II dose of 260 mg/m^2^ administered every 12 h on a continuous schedule in 28-day cycles, with clinical evaluation throughout to monitor response.	NCT05804227
	Sirolimus (AY-22989) mTOR Inhibitor	NF1-associated pNF in children and young adults ≥ 3 yrs	Phase II (Interventional, single-arm per stratum, multi-cohort design)	This study is designed to assess whether sirolimus can extend time to progression in progressive PN or induce radiographic reduction in non-progressing PN, while evaluating feasibility, toxicity, and drug exposure characteristics in individuals with NF1. Sirolimus does not shrink pNF, but it consistently slows their growth and shows biologic activity with acceptable toxicity.	Treatment consists of continuous twice-daily sirolimus administered in 28-day cycles with individualized dosing to therapeutic trough targets, alongside scheduled MRI volumetrics and clinical assessments to guide ongoing therapy.	NCT00634270
	Everolimus (RAD001) mTOR Inhibitor	NF1-associated pediatric LGG in children and young adults (3–22 yrs)	Phase II (Interventional, single-arm open-label)	This study is designed to evaluate whether daily everolimus can delay progression or induce shrinkage in NF1-associated low-grade gliomas while defining its safety profile and pharmacologic behavior. Across this and other studies, oral everolimus has not shown a significant reduction in lesion size [81].	Treatment consists of once-daily oral everolimus administered in 28-day continuous cycles at a dose of 5 mg/m^2^ (maximum 10 mg), beginning on study day 1 and continued for up to 12 cycles or until progression, toxicity, or completion of 48 weeks of therapy [82].	NCT01158651
	Imatinib (STI-571) RTKs Inhibitor	NF1-associated pNF (3–65 yrs)	Phase II (Interventional, single-arm, open-label)	This study is designed to determine whether daily imatinib can produce radiographic or clinical responses in NF1-associated pNF while characterizing toxicity and biomarker changes. Objective responses occurred in 17% of the intention-to-treat population and in 26% of those receiving imatinib for 6 months or longer, each defined as ≥20% PN volume reduction. Most toxicities were mild, including rash and edema, while serious events such as neutropenia, hyperglycemia, and hepatic enzyme elevation were uncommon and reversible [83].	Treatment consists of oral administration at 220 mg/m^2^ twice daily in children and 400 mg/m^2^ twice daily in adults, with dose reductions for toxicity.	NCT01673009
	Sorafenib (BAY 43-9006, Nexavar) RTKs Inhibitor	Pediatric Ras-driven tumors, specifically NF1-associated inoperable pNF in children and young adults (3–18 yrs)	Phase I (Interventional, dose-escalation, single-arm, open-label)	This study is designed to establish the maximum tolerated dose and characterize the safety and biologic activity of sorafenib in NF1-associated pNF.	Treatment consists of continuous twice-daily sorafenib in 28-day cycles, with clinical evaluation throughout to monitor response.	NCT00727233 [84]
	Cabozantinib (XL l84) RTKs Inhibitor	NF1-associated pNF in children (3–15 yrs)	Phase II (Interventional, single-arm, open-label)	This study is designed to evaluate whether cabozantinib can achieve meaningful volumetric reduction in NF1-associated pNF, while establishing its tolerability and pharmacokinetic profile in pediatric and adult patients. Cabozantinib met its primary endpoint, achieving partial responses in 42% of evaluable patients, with a median 15.2% tumor-volume reduction and no on-treatment progression, while secondary analyses demonstrated consistent safety, pharmacokinetic profiles, and improvements in pain and quality-of-life measures [85].	Cohort B (ages 3–15 yrs): 30 mg/m^2^ daily with escalation to 40 mg/m^2^ at cycle 3 if tolerated; reductions to 23–30 mg/m^2^ for toxicity.	NCT02101736
	Pexidartinib (PLX3397) RTKs inhibitor	NF1-associated pNF and MPNST in children and adults (3–35 yrs)	Phase I (Interventional, single-arm, open-label)	This study is designed to define the safety profile and recommended phase II dose of pexidartinib in children and young adults with refractory malignancies, including NF1 pnF and MPNST.	Therapy consists of once-daily oral dosing (125 mg capsules) in continuous 28-day cycles with escalation based on MTD and expansion at RP2D to evaluate toxicity, PK, and early signals of clinical activity.	NCT02390752
	Neoadjuvant nivolumab plus ipilimumab PD-1 inhibitor and CTLA-4 inhibitor	NF1-associated pre-malignant neurofibroma and MPNST in patients (12–100 yrs)	Phase I (Interventional, single-group, open-label, early-phase)	This study is designed to test the safety and feasibility of administering dual checkpoint inhibition before surgical resection of NF1-associated ANF or MPNST	Participants receive neoadjuvant combination immunotherapy with nivolumab and ipilimumab prior to standard-of-care management. Nivolumab is administered at 4.5 mg/kg intravenously every 3 weeks for 2 doses, together with ipilimumab 1 mg/kg intravenously every 3 weeks for 2 doses, with clinical evaluation throughout to monitor response.	NCT04465643
	ASTX727 (INQOVI, combination of cedazuridine and decitabine) Cytidine deaminase (CDA) inhibitor; DNA methyltransferase (DNMT) inhibitor;	PRC2-loss MPNST in adults or adolescents	Phase II (Interventional, single-arm, open-label, single-group)	This study is designed to evaluate the therapeutic activity, safety, and tolerability of oral ASTX727 in patients meeting eligibility criteria for hypomethylating-agent-based therapy, with additional assessment of hematologic and clinical responses.	The trial administers oral cedazuridine/decitabine once daily on days 1–5 of each 21-day cycle with pegfilgrastim support on day 7, with clinical evaluation throughout to monitor response.	NCT04872543

## Abbreviations

The following abbreviations are used in this manuscript:

NF1	Neurofibromatosis Type 1
cNFs	cutaneous neurofibromas
pNFs	plexiform neurofibromas
MPNSTs	malignant peripheral nerve sheath tumors
HGMD^®^	Human Gene Mutation Database
GAP	GTPase-activating protein
NLS	nuclear localization signal
CSRD	cysteine/serine-rich domain
TBD	tubulin-binding region
GRD	GAP-related domain
LRD	leucine-rich domain
PH	pleckstrin homology
CTD	C-terminal domain
SBR	syndecan-binding region
SynGAP	synaptic RasGAP
PAM	PI3K/AKT/mTOR
AC	adenylyl cyclase
GPCR	G protein-coupled receptors
5-HT6r	5-hydroxytryptamine receptor 6
LRPPRC	leucine-rich pentatricopeptide-repeat-containing protein
APP	amyloid precursor protein
CALMs	café-au-lait macules
LOH	loss of heterozygosity
PRC2	Polycomb Repressive Complex 2
AAV	adeno-associated virus
HSV	herpes simplex virus
CAR-T	chimeric antigen receptor T cell
oHSV	oncolytic herpes simplex virus
T-VEC	Talimogene laherparepvec
DLT	dose-limiting toxicities
MTD	maximum tolerated dose
CART	Antigen-specific cytokine-activated T cells
CTLs	cytotoxic T lymphocytes
DCvac	dendritic cell vaccine
LGG	low-grade glioma
rAAVs	recombinant adeno-associated viruses
hSCs	human Schwann cells
C10	C-terminal 10 AA of HRAS domain
C24	C-terminal 24 AA of KRAS4B
IF	Immunofluorescence
hNu	human nuclear antigen
RTKs	receptor tyrosine kinases
HDACis	histone deacetylase inhibitors
